# A Fast and Robust Extrinsic Calibration for RGB-D Camera Networks †

**DOI:** 10.3390/s18010235

**Published:** 2018-01-15

**Authors:** Po-Chang Su, Ju Shen, Wanxin Xu, Sen-Ching S. Cheung, Ying Luo

**Affiliations:** 1 Center for Visualization and Virtual Environments, University of Kentucky, Lexington, KY 40506, USA; wxbit0930@gmail.com (W.X.); sccheung@ieee.org (S.-C.S.C.); 2 Interactive Visual Media (IVDIA) Lab, University of Dayton, Dayton, OH 45469, USA; jshen1@udayton.edu; 3 Department of Computer Information Technology and Graphics, Purdue University Northwest, Hammond, IN 46323, USA; ying.luo@pnw.edu

**Keywords:** RGB-D camera, spherical object, camera network calibration, 3D reconstruction

## Abstract

From object tracking to 3D reconstruction, RGB-Depth (RGB-D) camera networks play an increasingly important role in many vision and graphics applications. Practical applications often use sparsely-placed cameras to maximize visibility, while using as few cameras as possible to minimize cost. In general, it is challenging to calibrate sparse camera networks due to the lack of shared scene features across different camera views. In this paper, we propose a novel algorithm that can accurately and rapidly calibrate the geometric relationships across an arbitrary number of RGB-D cameras on a network. Our work has a number of novel features. First, to cope with the wide separation between different cameras, we establish view correspondences by using a spherical calibration object. We show that this approach outperforms other techniques based on planar calibration objects. Second, instead of modeling camera extrinsic calibration using rigid transformation, which is optimal only for pinhole cameras, we systematically test different view transformation functions including rigid transformation, polynomial transformation and manifold regression to determine the most robust mapping that generalizes well to unseen data. Third, we reformulate the celebrated bundle adjustment procedure to minimize the global 3D reprojection error so as to fine-tune the initial estimates. Finally, our scalable client-server architecture is computationally efficient: the calibration of a five-camera system, including data capture, can be done in minutes using only commodity PCs. Our proposed framework is compared with other state-of-the-arts systems using both quantitative measurements and visual alignment results of the merged point clouds.

## 1. Introduction

When capturing static or dynamic scenes for different augmented or mixed reality applications, using multiple networked cameras has many advantages over a single camera. A single camera suffers from unintuitive, self-occluding hulls when capturing non-convex articulated 3D shapes like human bodies. The field of view and spatial resolutions of a single camera, especially depth cameras, are often limited. Simultaneous Localization and Mapping (SLAM) techniques [1,2,3,4] with a moving camera can be used to capture a large static environment, but does not work for dynamic scenes. On the other hand, using a stationary camera network can address the limitations in both field of view and dynamic scene capturing. There is already a large body of work using color camera networks for various types of vision processing [5,6,7]. Camera networks based on depth sensors such as Time-of-Flight (ToF) or structured light cameras, however, are not as well-explored. Earlier depth cameras suffer from high measurement noise and low spatial resolution. Due to the recent success of low-cost commodity depth cameras such as Kinect and Xtion Pro Live, there have been significant improvements in performance, thanks to better sensing technology and the addition of a companion high-definition color camera [8,9,10]. In fact, utilizing a pair of color and depth cameras has solved a number of challenging problems. For example, missing depth values and depth misalignment on planar surfaces can be recovered by exploiting the co-located color and depth information [11,12]. It is natural to extend from a single RGB-D camera to a network of RGB-D cameras, which are beneficial to a myriad of applications including 3D model rendering, body pose tracking and understanding [13,14,15].

One of the prerequisites in using multiple RGB-D cameras for these applications is to calibrate individual cameras into a unified coordinate system. There are three main challenges to this task: first, the captured data from depth cameras often have missing and noisy measurements, particularly on transparent or specular surfaces, and near depth discontinuities. These imperfections can greatly deteriorate the accuracy of the geometric alignment. Second, the sparsity of cameras makes it difficult to locate common scene features needed for calibration across disparate camera views. Adjacent camera views may share more scene features, but even small alignment error between adjacent views could accumulate when it is extrapolated to the entire network. Finally, as multiple cameras are often cumbersome to setup and maintain, it is highly desirable to make the calibration procedure robust and easily adaptable to any changes in the camera placement. There have been a number of recent works on RGB-D network calibration [14,16,17,18], but as we shall point out in Section 2, these approaches are either impractical or prone to errors.

In this paper, we propose a fast and robust algorithm for calibrating a network of multiple RGB-D cameras. Using only commodity hardware, the entire calibration process of a five-camera network takes only minutes to complete. Our algorithm uses a spherical object for calibration. An effective sphere-fitting algorithm is first used to identify the moving locations of the sphere center in both the color and depth images. An initial estimate of the extrinsic parameters is then obtained based on the corresponding locations across different views. In the final step, the extrinsic parameters are further refined using a simultaneous optimization of the entire network. The main contributions of our work are as follows:

1. Unlike other approaches that rely on planar calibration objects, our usage of a spherical object overcomes the problem of limited scene features shared by sparsely-placed cameras. Specifically, the location of the sphere center can be robustly estimated from any viewpoint as long as a small part of the sphere surface can be observed.
2. Rigid transformation is typically used to represent camera extrinsic calibration and has been shown to be optimal for the pinhole camera model. However, real cameras have imperfections, and a more flexible transformation could provide higher fidelity in aligning 3D point clouds from different cameras. We systematically compare a broad range of transformation functions including rigid transformation, intrinsic-extrinsic factorization, polynomial regression and manifold regression. Our experiments demonstrate that linear regression produces the most accurate calibration results.
3. In order to provide an efficient calibration procedure and to support real-time 3D rendering and dynamic viewpoints, our proposed algorithm is implemented in a client-and-server architecture where data capturing and much of the 3D processing are carried out at the clients.

This paper is an extension of our previous work in [19]. In this work, we further provide a thorough comparison of different view transformations and incorporate a simultaneous optimization procedure in refining the results. The rest of the paper is organized as follows. Section 2 reviews recent literature on the camera calibration problem. In Section 3, we describe in detail our proposed system, which includes sphere center detection, pairwise camera calibration and the simultaneous optimization for the camera network. The setup for our experiments and the evaluation results can be found in Section 4. We conclude the paper in Section 5.

## 2. Related Work

Extrinsic calibration requires a calibration object visible to different cameras in order to establish correspondences. For color camera calibration, commonly-used calibration objects include planar checkerboards [5,20,21,22], laser pointers [23,24,25], circular patterns [26,27], planar mirrors [28] and other custom-made objects [29]. None of these calibration objects work for depth sensors as they rely on distinctive colors or texture patterns that are not observable by depth sensors. Additionally, they require a dense distribution of a camera network to obtain accurate camera extrinsics. As such, their calibration procedures tend to be time-consuming and are unable to restore the calibration rapidly in a dynamic capture environment, where cameras may be added, moved or removed. Instead, objects with significant depth variations need to be used to calibrate depth sensors. For example, a planar calibration pattern with holes were used in [30]. Planar objects were also used by Herrera et al. who utilized the four corners of the calibration plane [31]. Liu et al. instead used a moving stick with one end fixed for RGB-D camera calibration [32]. A common drawback of these approaches is that the sharp depth edges along these objects usually have significant measurement noise on the depth images. Such noise can lead to erroneous correspondences across different views.

In [33], the authors of [31] improved their earlier scheme by using the planarity constraint defined based on the corners of a checkerboard plane. The use of planar, instead of point, features alleviates the problem associated with depth discontinuities. Similar approaches could also be found in [34,35], both of which used planarity constraints to detect the correspondences between the depth images. However, the calibration accuracy is still poor due to the low spatial resolution of depth cameras. To improve the accuracy, Shim et al. used the corresponding 3D positions, rather than 2D features, to optimally calibrate multiple RGB-D cameras [9]. Specifically, they identified the two major sources of depth measurement error to be the changes in scene depth and the amount of captured infrared light. Based on these two factors, they constructed an error model to optimize the calibration results. On the other hand, the authors did not address the issue of limited common scene features when the cameras are sparsely spaced.

Calibrations without using any specialized reference objects or patterns have also been studied [36,37,38]. In [36], a silhouette extracted from a person was used for calibration. In [37], Carrera et al. calibrated a robotic camera platform by detecting invariant SURF feature correspondences across different views. In [38], the extrinsic parameters were estimated based on point correspondences established from the unstructured motion of objects in the scene. These methods typically have lower precision than those based on reference objects due to imprecise knowledge of the unknown scene features, which can lead to erroneous correspondences from different viewpoints. In [39], Li et al. proposed a method to calibrate multiple cameras based on users’ joint positions. The calibration process can be accomplished by aligning skeleton data across different camera views. However, their system has difficulty in fusing noisy skeleton data from the wide-baseline camera network setup.

Besides calibration objects, another key difference between RGB-D cameras and color cameras is the availability of both color and depth information in RGB-D cameras. Appropriate fusion of multiple data channels can potentially achieve more accurate depth measurements and extrinsic calibration [8,10,40]. Prasad et al. first demonstrated depth resolution enhancement through color and depth registration by using a novel system with a 2D sensor, a 3D sensor and an image multiplier [40]. In [8], reference depth images generated by a pair of stereo cameras were used to calibrate a Time-of-Flight (ToF) depth sensor. The depth image quality can also be improved by utilizing both active and passive depth measurements. Hansard et al. used 3D projective transformation to calibrate both the ToF and color cameras [10]. The geometric relation could then be found by aligning range images with parallax reconstructions.

With the advent of low-cost commodity RGB-D cameras, there are now software libraries that can easily align the depth image to the color image for an RGB-D camera [41]. However, a common assumption of all these methods is a close baseline among different cameras so that the transformation among different views can be easily accomplished. Based on [41], the works in [14,16,18] calibrated an RGB-D camera network to reconstruct 3D objects. Nevertheless, none of them can accurately reconstruct an entire 3D scene including static background and dynamic foreground as they utilized the checkerboard and iterative closest point algorithm to align dense point clouds of foreground objects. The reconstructed background scenes would be misaligned based on their proposed methods.

Increasingly, the topic of calibration of wide-area networks of sparsely-spaced cameras has been investigated [7,42,43,44], though the majority of the techniques require special equipment and image features. Kuo et al. used GPS position and images taken by mobile devices to calibrate a fixed camera in a camera network [7]. Ly et al. utilized the image of lines to improve the calibration results for multiple cameras with only partially overlapping fields of view [42]. In [43], an active self-calibration of a multi-camera system scheme was proposed to solve the problem of non-overlapping views and occlusion by automatically rotating and zooming each camera. A probabilistic model was used to find the appropriate relative pose during extrinsic calibration. In [44], the authors used large planer scenes such as the floor or ceiling to calibrate cameras with disparate views. The use of pan-tilt-zoom cameras or special scene features limits the types of applications where these techniques can be deployed.

While we were the first to propose using spherical objects in calibrating multiple RGB-D camera networks [19], there are other works, such as [17,45,46,47,48], that also utilized spherical objects for RGB-D network calibration. However, our current work goes beyond accurate calibration for foreground object reconstruction in [45,46] to reconstruction of the entire environment through flexible transformations and global optimization. In [47,48], the authors have shown that using a spherical object for calibration could produce better results than using the traditional checkerboard as in [35]. However, the comparisons were done only for cameras separated by a narrow baseline. For sparse camera networks, Ruan et al. used a spherical object and estimated the location of the sphere center for extrinsic calibration of multiple depth cameras [17]. However, the sphere detection was not very robust because color information was not used. Furthermore, as the cameras were not time-synchronized, the technique was labor intensive as the sphere needed to be physically moved and affixed to different locations in order to capture enough data for calibration. In [19], we independently proposed an RGB-D camera network calibration scheme based on sphere center detection. Using both the color and depth channels, we developed an automatic noise removal algorithm to robustly identify the sphere and estimate its center location. As our cameras were time-synchronized, a user could simply waive the sphere in the environment once, and the data collection process would be done. A drawback of [19] is its reliance on the simplistic rigid transformation-based pairwise camera registration, which is inadequate for non-pinhole cameras and can lead to error accumulation. In this paper, we extend the scheme in [19] by first using a more flexible view transformation function to minimize error in registration and then introduce a simultaneous optimization framework to further refine the extrinsic parameters. Comparison of the proposed scheme with our earlier work and other state-of-the-art schemes discussed here can be found in Section 4.

## 3. Proposed Method

**Figure 1:**
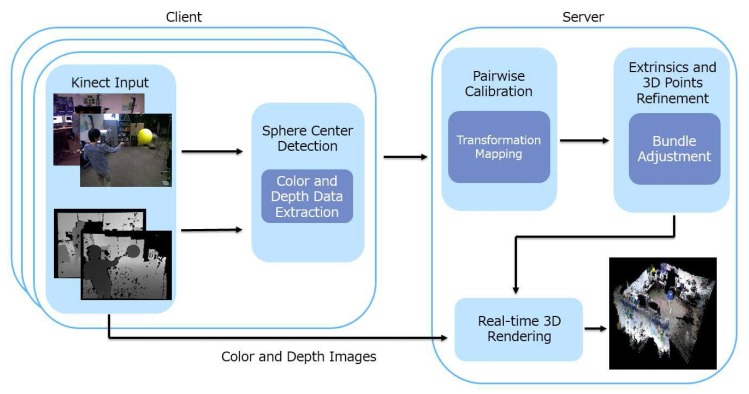
This figure provides an overview of our RGB-D camera calibration framework for real-time 3D rendering in a client-server distributed architecture. On the client side, each of the Kinect camera clients produces a pair of color and depth images. The sphere center detection module uses these raw data to estimate the location of the sphere center. During calibration, the estimates are sent to the server, which produces first a rough estimate of the extrinsic camera matrices and the sphere center locations in the world coordinate system. The results are then refined by a simultaneous optimization process to produce optimal extrinsic matrices, which are used to produce real-time rendering results.

The block diagram in Figure 1 shows the basic architecture and data flow of our proposed framework. Each RGB-D camera is controlled by a client process. The server process, which can be run on the same computer as the clients or a separate computer on the network, collects all necessary information from the clients to compute the extrinsic parameters. All the client processes and the server process are time-synchronized using the Network Time Protocol (NTP) with a time drift of less than 4 milliseconds [49]. While the accuracy of the extrinsic parameters could be measured with respect to ground truth data, the ultimate test is how well they can contribute to the 3D reconstruction of a real-world scene beyond the ground truth set. As such, our architecture is designed with this goal in mind and supports real-time 3D rendering: color and depth images are compressed and streamed to the server, which can use the previously-computed extrinsic parameters to perform 3D reconstruction in real time. Our framework applies to camera networks consisting of co-located depth-color cameras such as Kinect cameras. The main functional blocks in Figure 1 are as follows:

- Sphere center detection: The 3D locations of the center of a moving sphere are estimated from the color and depth images. They are used as visual correspondences across different camera views. There are two reasons for choosing a sphere as a calibration object. First, it is suitable for a wide baseline: any small surface patch on the sphere is sufficient to estimate the location of its center. As such, two cameras capturing different sides of the sphere can still use the sphere center as a correspondence. Second, instead of using the error-prone point or edge features as correspondences, depth measurements of the sphere surface are mostly accurate, and the spherical constraint can be used to provide a robust estimate of the center location. This step is independently executed at each camera client. The details of the procedure can be found in Section 3.2.
- Pairwise calibration: To provide an initial estimate of the extrinsic parameters of each camera, we perform pairwise calibration to find the view transformation function from each camera to an arbitrarily-chosen reference coordinate system. The server receives from each client the estimated sphere center locations and the associated time-stamps. Correspondences are established by grouping measurements from different cameras that are collected within the time synchronization error tolerance. Then, a system of equations with all correspondences as data terms and parameters of the view transformations as unknowns are solved at the server to provide an initial guess of the transformation functions. Details of this step can be found in Section 3.3.
- Simultaneous optimization: The estimated view transformations are then used to bootstrap a pseudo bundle adjustment procedure. This procedure simultaneously adjusts all the extrinsic parameters and the true 3D locations of the sphere center so as to minimize the sum of 3D projection errors across the entire network. Details of this step can be found in Section 3.4.

### 3.1. Problem Formulation

Before we delve into the details of each component, this section formulates the problem of the extrinsic calibration of RGB-D camera network and defines all the symbols used in this paper. An RGB-D sensor consists of a color camera and a depth camera. We first formalized the color camera projection process. Using the coordinate system at the optical center of the color camera as a reference, we denote a 3D scene point as $X_{c}={\left[X_{c},Y_{c},Z_{c},1\right]}^{T}$. The subscript *c* indicates the usage of the color camera’s coordinate system. The color camera project process is modeled by a 3×3 camera projection matrix $K_{c}$ and a scalar distortion function $L_{c}\left(\cdot \right)$. Specifically, $X_{c}$ is projected onto the image coordinate $x_{c}$ on the color camera plane as follows:

(1) $$x_{c}=K_{c}\cdot L_{c}\left(∥\left[\begin{array}{c} X_{c}/Z_{c}\\ Y_{c}/Z_{c} \end{array}\right]∥\right)\left[\begin{array}{c} X_{c}/Z_{c}\\ Y_{c}/Z_{c}\\ 1 \end{array}\right]$$

The camera matrix $K_{c}$ is defined as follows:

(2) $$K_{c}=\left[\begin{array}{ccc} f_{x} & \gamma & o_{x}\\ 0 & f_{y} & o_{y}\\ 0 & 0 & 1 \end{array}\right]$$

based on the intrinsic parameters of the camera including the focal lengths $\left(f_{x},f_{y}\right)$, the principal point $\left(o_{x},o_{y}\right)$ and the skew factor γ. $L_{c}\left(\cdot \right)$ is a scalar distortion function that models the radial distortion of the lens, typically expressed as a sixth-degree polynomial [50]. Methods to obtain these intrinsic camera parameters are well documented [20].

The depth camera model projects the 3D scene point $X_{d}={\left[X_{d},Y_{d},Z_{d},1\right]}^{T}$ with respect to its local coordinate system to two components: the 2D image coordinates $x_{d}={\left[x_{d},y_{d},1\right]}^{T}$ and the depth measurement $z_{d}$. For the 2D image coordinates, the projection process is similar to that of the color camera as described in Equation (1):

(3) $$x_{d}=K_{d}\cdot L_{d}\left(∥\left[\begin{array}{c} X_{d}/Z_{d}\\ Y_{d}/Z_{d} \end{array}\right]∥\right)\left[\begin{array}{c} X_{d}/Z_{d}\\ Y_{d}/Z_{d}\\ 1 \end{array}\right]$$

with its own camera matrix $K_{d}$ and distortion function $L_{d}$. The depth measurement is related to the actual depth based on the following model:

(4) $$z_{d}=\frac{1-\alpha_{1}Z_{d}}{\alpha_{0}Z_{d}}$$

where $\alpha_{0}$ and $\alpha_{1}$ are the parameters that correct the depth measurement [51]. To fuse the color and depth information, the color camera and the depth camera need to be calibrated in order to obtain the transformation $P_{d}$ between the two coordinate systems:

(5) $$X_{c}=P_{d}X_{d}$$

$P_{d}$ is pre-computed using the method in [52].

Consider a network of *m* RGB-D cameras $\{C_{1}$, $C_{2}$, …, $C_{m}\}$. The goal of the extrinsic calibration is to transform between the local coordinate system of each camera and an arbitrarily-chosen world coordinate system. Without loss of generality, we choose the coordinate system of the color camera $C_{1}$ to be our world coordinate system. To allow a broad range of extrinsic transformations, we consider the following formulation of the mapping between the 3D point $X_{w}$ in world coordinates to the 3D point $X_{d}^{\left(j\right)}$ in the *j*-th local depth camera coordinates:

(6) $$h\left(X_{d}^{\left(j\right)}\right)=P_{j}X_{w}\;\;\;\mathrm{for}\;j=1,\ldots ,m.$$

$P_{j}$ is the extrinsic matrix for the *j*-th depth camera and h(·) is a data-independent feature mapping that can introduce higher order terms to provide a potentially better fit of the data. The specific types of $P_{j}$ and h(·) tested in this paper are described in Section 3.3. The usages of Equation (6) in analysis and synthesis are different. During the analysis stage, we have multiple observations $X_{d}^{\left(j\right)}$ from different cameras of an unknown 3D point $X_{w}$. The goal is to estimate ${P_{j}}^{-1}h\left(\cdot \right)$ so as to minimize the overall discrepancies after projecting the image points onto the same world coordinate system. During the synthesis stage, we reverse the above process by using the estimated ${P_{j}}^{-1}h\left(\cdot \right)$ to project a known 3D point $X_{w}$ onto each local coordinate system. If the mapping ${P_{j}}^{-1}h\left(\cdot \right)$ is not invertible, its Moore–Penrose pseudoinverse, denoted as $h^{†}\left(P_{j}\cdot \right)$, will be used. For example, we can compute the color information by relating the local 3D point $X_{c}^{\left(j\right)}$ in the *j*-th color camera coordinates to $X_{w}$ using the following formula:

(7) $$X_{c}^{\left(j\right)}=P_{d}^{\left(j\right)}h^{†}\left(P_{j}X_{w}\right)\;\;\;\mathrm{for}\;j=1,\ldots ,m.$$

Equations (1), (3), (4), (6) and (7) altogether describe the relationship between an image point $\left(x_{c}^{\left(j\right)},x_{d}^{\left(j\right)},z_{d}^{\left(j\right)}\right)$ in each of the RGB-D cameras and a 3D scene point $X_{w}$ in the world coordinate system. The problem of extrinsic calibration can now be formulated as follows: using multiple $X_{w}$ and their corresponding camera image points $\left\{\left(x_{c}^{\left(1\right)},x_{d}^{\left(1\right)},z_{d}^{\left(1\right)}\right),\ldots ,\left(x_{c}^{\left(m\right)},x_{d}^{\left(m\right)},z_{d}^{\left(m\right)}\right)\right\}$ to optimally compute the extrinsic matrices $P_{j}$ for j=1,2,3,…,m.

### 3.2. Sphere Detection by Joint Color and Depth Information

**Figure 2:**
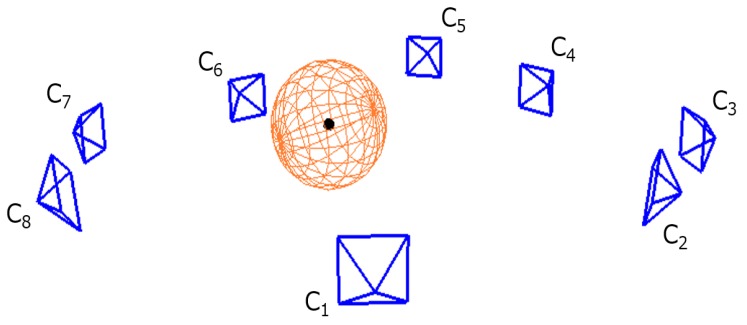
Sphere center detection in a camera network.

**Figure 3:**
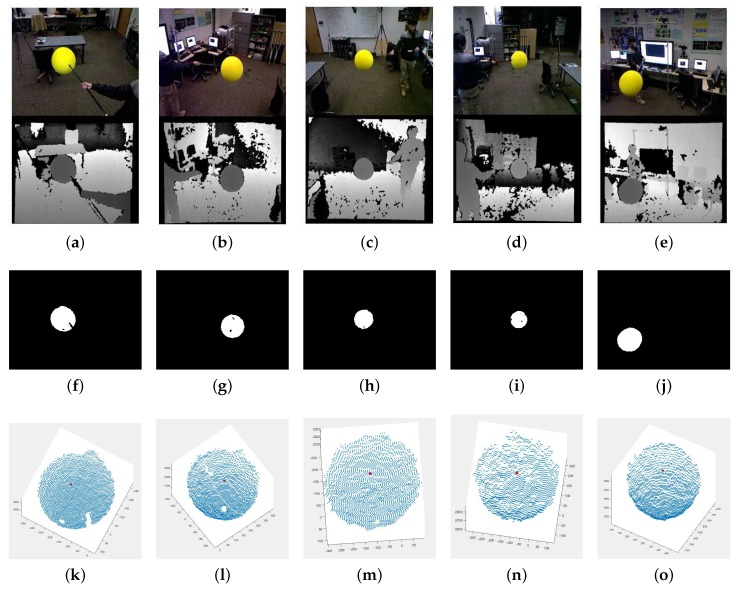
Sphere center detection: each column shows the process at a different camera in the network. The top row images (**a**–**e**) are the input RGB and depth images. The middle row images (**f**–**j**) show the results of detected sphere regions, and the bottom row images (**k**–**o**) represent the initial detected spheres in 3D with the red dots indicating the estimated sphere centers. Depending on the perspective view of the virtual camera, the sphere center may not be at the center of the detected sphere.

The prerequisite for solving the extrinsic calibration problem as described in Section 3.1 is to establish the correspondence between an image point from each camera and a 3D point in the physical space. Our proposed system uses the center of a spherical calibration object as the target 3D point for calibration. Figure 2 illustrates a sphere in the camera network, and Figure 3 shows our sphere detection process. While the sphere center is not directly visible to any camera, the spherical constraint implies that the observation of a reasonably-sized surface patch from any direction can be used to deduce the location of the center. In this section, we describe the algorithm in identifying the calibration object and estimating its center from the captured color and depth images.

To facilitate the detection of the sphere in the color channel, it is painted with a highly distinctive color (see the top row of Figure 3). To minimize the effect of varying illumination, we first convert the RGB image into HSV color space and detect the specific color using a pre-trained Gaussian mixture model classifier in the hue-saturation space. The real-time detection of the sphere is further aided by using a simple background subtraction and focusing the color search within the foreground region.

The detected color pixels of the sphere are then mapped to the corresponding depth pixels based on the intrinsic alignment between the two modalities as stated in Equation (5). Combining the spatial coordinates and the depth value, we can invert Equations (3) and (4) to obtain the local 3D coordinates of the detected sphere surface points. As pointed out in Section 1, depth measurements could be quite noisy. In addition, the IR interference between adjacent Kinect cameras can significantly degrade the depth measurements. The interference is due to neighboring structured light sensors projecting IR patterns in the same spectrum, thereby affecting the depth estimation at each camera. Many solutions have been proposed in the literature, including mounting the camera on a vibrating platform [53,54], using mechanical shutters to periodically block individual cameras [55] and software denoising techniques [56]. However, such an interference primarily affects the overlapping visual fields, which in our case are relatively small. Our camera configuration is designed to enlarge the coverage and adjacent cameras are usually kept at 90–180 degree angles where the interference is relatively minor [57]. In order to obtain a robust estimate of the center location based on these noisy measurements, we apply a RANSAC procedure by iteratively identifying all the 3D points that satisfy the surface equation of a 3D sphere of a known radius $\bar{r}$ [58]. While we use the known radius to increase robustness, the knowledge of it is not strictly necessary as the procedure is often accurate enough without the extra constraint. We compute the sphere equation, parameterized by $A_{1},A_{2},A_{3}$ and $A_{4}$, by carrying out the following constrained optimization:

(8) $$\min_{A_{1}\cdots A_{4}}\sum_{k}\left(x_{k}^{2}+y_{k}^{2}+z_{k}^{2}+A_{1}x_{k}+A_{2}y_{k}+A_{3}z_{k}-A_{4}\right)$$

subject to the constraint:

(9) $$\left|\;\sqrt{\left(A_{1}^{2}+A_{2}^{2}+A_{3}^{2}\right)/4-A_{4}}-\bar{r}\;\right|\leq \epsilon$$

where ϵ is a pre-defined error tolerance in the radius measurement. The estimated sphere center is given by $\left(-A_{1}/2,-A_{2}/2,-A_{3}/2\right)$. This estimation is highly robust in our setup for a number of reasons. First, the noisy depth measurements tend to concentrate around the edge of the sphere. However, this has little effect on the estimation of the sphere center location as it is an isotropic quantity. Second, we have chosen a large enough sphere (radius > 100 mm in a 5 m × 5 m room) so that the RANSAC procedure typically retains more than a thousand pixels per camera frame for the estimation. Even with a fair amount of occlusion, we have more than sufficient data points to solve for the optimization problem, which has only 4 degrees of freedom. The initial detected spheres in 3D with the estimated sphere centers are shown in Figure 3k–o. Repeating the same procedure for *n* video frames, we obtain the trajectory $\left\{c_{1},\ldots ,c_{n}\right\}$ of the estimated sphere centers in the local 3D space.

### 3.3. Extrinsic Calibration between Pairwise Cameras

**Figure 4:**
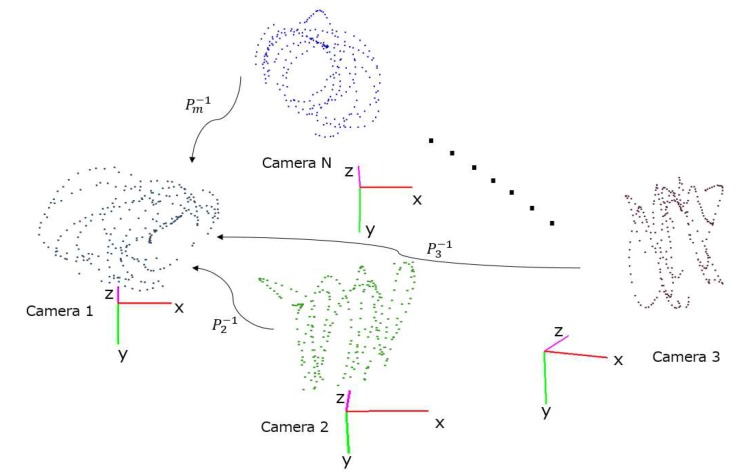
Pairwise calibration: Camera 2 to Camera N are aligned to the reference coordinate (Camera 1).

After the locations of the moving sphere center are detected at each camera, we can use them as correspondences to estimate the extrinsic parameters between each camera and the reference camera frame as illustrated in Figure 4. The focus on a pair of camera simplifies the optimization, but is likely to be suboptimal. As such, this step only produces an initial estimate of the extrinsic parameters, which will be later refined in Section 3.4. While all cameras are time-synchronized, the sphere may not be simultaneously visible to both cameras in question. Thus, the first step is to filter out those frames in which the sphere is visible to one, but not the other. We denote the filtered, time-synchronized trajectories of sphere center locations in local 3D coordinates at camera pair $C_{r}$ and $C_{q}$ as $\left\{C_{1}^{\left(r\right)},C_{2}^{\left(r\right)},\ldots ,C_{n}^{\left(r\right)}\right\}$ and $\left\{C_{1}^{\left(q\right)},C_{2}^{\left(q\right)},\ldots ,C_{n}^{\left(q\right)}\right\}$. To keep the calibration effort low, the number of calibration data points could be quite small, so the challenge is to use a flexible transformation that can generalize well to the entire scene based the limited training data. Existing approaches almost exclusively focus on using rigid transformation, but it is unclear if there are other types of transformations that might be able to produce better results. As such, we have experimentally tested a number of different transformations, which are reviewed in the following subsections.

#### 3.3.1. Rigid Transformation

The six degrees of freedom rigid transformation is commonly used to describe a relative camera pose in 3D space. For the camera pair $\left(C_{q},C_{r}\right)$, the transformation is determined by a rotation matrix $R^{\left(qr\right)}$ parameterized by the three rotation angles $\theta_{x},\theta_{y}$ and $\theta_{z}$ and a translation vector $t^{\left(qr\right)}={\left[t_{x},t_{y},t_{z}\right]}^{T}$ between the two camera centers. Putting them in the form of Equation (7) with $C_{r}$ as the world (reference) coordinate system, we have h(·) as the identity function and the extrinsic matrix as:

(10) $$P_{q}^{-1}=\left(\begin{array}{cc} R^{\left(qr\right)} & t^{\left(qr\right)}\\ 0 & 1 \end{array}\right)$$

To compute each unknown parameter, we require at least n≥3 point correspondences. Our goal is to find $R^{\left(qr\right)}$ and $t^{\left(qr\right)}$ that minimize the following cost function:

(11) $$J_{RT}\left(R^{\left(qr\right)},t^{\left(qr\right)}\right)=\sum_{i=1}^{n}{∥C_{i}^{\left(r\right)}-P_{q}^{-1}C_{i}^{\left(q\right)}∥}^{2}$$

Due to the orthogonality constraint on the rotation matrix $R^{\left(qr\right)}$, we use the least-squares-based method in [59] by first computing the covariance matrix as follows:

(12) $$A=\sum_{i=1}^{n}\left[\left({\bar{C}}^{\left(q\right)}-C_{i}^{\left(q\right)}\right)\cdot {\left({\bar{C}}^{\left(r\right)}-C_{i}^{\left(r\right)}\right)}^{T}\right]$$

where ${\bar{C}}^{\left(q\right)}=\frac{1}{n}\cdot \sum_{i=1}^{n}C_{i}^{\left(q\right)}$ and ${\bar{C}}^{\left(r\right)}=\frac{1}{n}\cdot \sum_{i=1}^{n}C_{i}^{\left(r\right)}$ are the respective centroids of the two correspondence sets. Using singular value decomposition $A={\mathrm{USV}}^{T}$, we can compute the rotation matrix as $R^{\left(qr\right)}={\mathrm{VU}}^{T}$ and $t^{\left(qr\right)}={\bar{C}}^{\left(q\right)}-{\bar{C}}^{\left(r\right)}$.

#### 3.3.2. Polynomial Regression

The rigid transformation is sufficient if all sources of intrinsic distortion have been fully compensated. In practice, there are always residual error, and a more flexible regression model could further minimize the error without overfitting. In this section, we focus on *d*-degree polynomial transformation $F^{\left(qr\right)}\left(\cdot \right)$ to map $C_{i}^{\left(q\right)}$ to $C_{i}^{\left(r\right)}$ for i=1,2,…,n. We can parameterize the polynomial fitting problem by treating $F^{\left(qr\right)}$ as a matrix multiplication with the extrinsic matrix $P_{q}^{-1}$, again treating $C_{r}$ as the world frame, after a feature mapping function $h_{d}\left(\cdot \right)$. The overall cost function to be minimized is as follows:

(13) $$J_{PR}\left(P_{q}^{-1}\right)=\sum_{i=1}^{n}{∥C_{i}^{\left(r\right)}-P_{q}^{-1}h_{d}\left(C_{i}^{\left(q\right)}\right)∥}^{2}$$

$h_{d}\left(\cdot \right)$ expands an input 3D point into the products of all cross terms with up to *d* coordinates. For example, in the case d=2, $h_{2}\left(\cdot \right)$ is as follows:

(14) $$h_{2}\left({\left[x\;\;y\;\;z\;1\right]}^{T}\right)={\left[x^{2}\;\;y^{2}\;\;z^{2}\;\;xy\;\;xz\;\;yz\;\;x\;\;y\;\;z\;\;1\right]}^{T}$$

The corresponding extrinsic matrix $P_{q}^{-1}$ would be a 4×10 matrix. This matrix has 30 degrees of freedom after removing redundancy based on the use of homogeneous coordinates. While a regression function with a higher degree can fit the calibration data better, it might have problems generalizing to unseen data, especially outside the vicinity of the sphere trajectory. This problem can be addressed by cross-validation, and we will evaluate and compare regression functions of different degrees in Section 4.

#### 3.3.3. Manifold Alignment

Even without overfitting, non-rigid transformations can produce non-Euclidean artifacts that can significantly degrade the quality of the 3D reconstruction. As such, it is important to preserve as much as possible the metric relationship within the data. Manifold alignment [60], unlike rigid body transformation and polynomial regression, can align correspondences across datasets, while preserving metric structures within each individual dataset. For camera calibration, its flexibility can potentially model the alignment better than rigid transformation, while preserving the Euclidean relationship better than polynomial regression. In this paper, we adapt the feature-level alignment in [60] for our camera calibration problem. Given two valid 3D trajectories at cameras $C_{r}$ and $C_{q}$, the mapping functions $\left(F^{\left(r\right)},F^{\left(q\right)}\right)$ can register the points in the manifold space by minimizing the following cost function:

(15) $$\begin{array}{cc} J_{MA}\left(F^{\left(r\right)},F^{\left(q\right)}\right) & =\mu \sum_{i=1}^{n}∥F^{\left(r\right)}C_{i}^{\left(r\right)}-F^{\left(q\right)}C_{i}^{\left(q\right)}{∥}^{2}+\sum_{i=1}^{n}\sum_{j=1}^{n}W_{r}^{i,j}{∥F^{\left(r\right)}C_{i}^{\left(r\right)}-F^{\left(r\right)}C_{j}^{\left(r\right)}∥}^{2}\\ & +\sum_{i=1}^{n}\sum_{j=1}^{n}W_{q}^{i,j}{∥F^{\left(q\right)}C_{i}^{\left(q\right)}-F^{\left(q\right)}C_{j}^{\left(q\right)}∥}^{2} \end{array}$$

The first term of Equation (15) is the alignment cost between the two trajectories. The second and the third terms attempt to preserve the local metric relationship by incorporating the similarity measurements $W_{r}^{i,j}$ and $W_{q}^{i,j}$. Specifically, $W_{r}^{i,j}=\exp(-∥C_{i}^{\left(r\right)}-C_{j}^{\left(r\right)}{∥}^{2})$ and $W_{q}^{i,j}=\exp(-∥C_{i}^{\left(q\right)}-C_{j}^{\left(q\right)}{∥}^{2})$. μ is an empirical parameter to balance the two parts of the cost function.

To map the manifold alignment representation to our extrinsic matrix representation, it is easy to see that $P_{q}^{-1}={\left(F^{\left(r\right)}\right)}^{-1}F^{\left(q\right)}$ with an identity feature mapping. To ensure both $F^{\left(r\right)}$ and $F^{\left(q\right)}$ are invertible, the formulation in [60] also incorporates a regularization constraint to enforce a constant volume after the alignment. Unlike rigid transformation or polynomial regression, *m* invocations of pairwise manifold alignment with the reference camera will produce *m* different transformations at the reference camera. In order to produce just one transformation at the reference frame, we modify the cost function (15) so that the same transformation is used to simultaneously minimize the error with respect to every other camera.

### 3.4. Simultaneous Optimization

In the final stage, we jointly refine all the extrinsic parameters estimated from the previous steps to produce the simultaneously optimal extrinsic parameters. Our simultaneous optimization algorithm is based on Bundle Adjustment (BA) [61], which has been widely used in many 3D reconstruction applications. The goal of bundle adjustment is to simultaneously adjust the camera parameters and 3D points to minimize the overall projection error between the observed and expected locations of the 3D points. In the original formulation of [61], BA was carried out based on the estimated 3D points and their 2D projections as follows:

(16) $$\min_{P_{j},X_{i}}\sum_{i=1}^{n}\sum_{j=1}^{m}v_{ij}d{\left(f\left(P_{j},X_{i}\right),{\tilde{x}}_{ij}\right)}^{2}$$

where *m* and *n* are the total number of cameras and 3D scene points, respectively. The function *f* denotes the relation that maps 3D point $X_{i}$ in world coordinates to 2D image pixel $x_{ij}$ by the corresponding projection matrix $P_{j}$. The variable $v_{ij}\in \left\{0,1\right\}$ indicates whether the point is visible by camera *j*. The function *d* denotes a distance function on the camera plane.

For our problem, we are interested in minimizing distance errors in 3D space instead of in 2D, and the obtained optimal extrinsic parameters will be used for our real-time 3D rendering. We assume that the intrinsic parameters of all cameras are known. The input of for this stage are the *m* sequences of *n* 3D sphere center locations from the *m* RGB-D cameras: $\left\{C_{1}^{\left(j\right)},C_{2}^{\left(j\right)}\;\cdots ,C_{n}^{\left(j\right)}\right\}$ for j=1,2,…,m. The goal is to find the *n* “true” 3D points $\left\{C_{1},C_{2}\;\cdots ,C_{n}\right\}$ and the optimal extrinsic matrices $P_{j}$ for j=1,2,…,m that transform these 3D points to the *m* observed sphere center sequences. Our pseudo-bundle adjustment equation can be written as follows:

(17) $$\min_{P_{j},C_{i=1,\ldots ,n}}\sum_{i=1}^{n}\sum_{j=1}^{m}v_{ij}∥h^{†}\left(P_{j}C_{i}\right)-C_{i}^{\left(j\right)}{∥}^{2}$$

Different from the classical BA, our formulation uses the 3D Euclidean distance in the local camera coordinate system. The minimization problem (17) is non-linear, and the standard approach is to use the Levenberg–Marquardt (LM) algorithm [62,63], which is an iterative procedure to find a local minimum of a cost function. At the *t*-th iteration step, our adapted LM procedure first computes an estimate of ${\left[C_{i}\right]}_{t}$ for i=1,2,…,n based on averaging the projections of the data points onto the world frame using the estimated ${\left[P_{j}\right]}_{t-1}$:

(18) $${\left[C_{i}\right]}_{t}=\frac{1}{\sum_{j=1}^{m}v_{ij}}\sum_{j=1}^{m}v_{ij}{\left[P_{j}\right]}_{t-1}^{†}h\left(C_{i}^{\left(j\right)}\right)$$

Then, LM updates the estimates of the extrinsic matrices as follows:

(19) $${\left[P_{j}\right]}_{t}={\left[P_{j}\right]}_{t-1}+\Delta_{t}^{\left(j\right)}$$

where $\Delta_{t}^{\left(j\right)}$ is determined based on a combination of steepest-descent and Gaussian–Newton methods in minimizing the cost function in (17), but fixing $C_{i}={\left[C_{i}\right]}_{t}$ for i=1,2,…,n. The iteration continues until the reduction in the cost function becomes negligible. The details of the LM procedure can be found in [63].

## 4. Experiments

The proposed algorithm is agnostic about the type of depth-sensing technologies, may that be stereo, structured-light or time-of-flight RGB-D cameras. For concreteness, we have chosen to use Microsoft Kinect v.1 structured-light cameras to capture all color and depth images in the experiments. They are inexpensive, which is an important consideration to build a large camera network. In addition, a recent study has shown that structured-light cameras provide a good price and performance tradeoff among different types of depth sensors [64].

**Figure 5:**
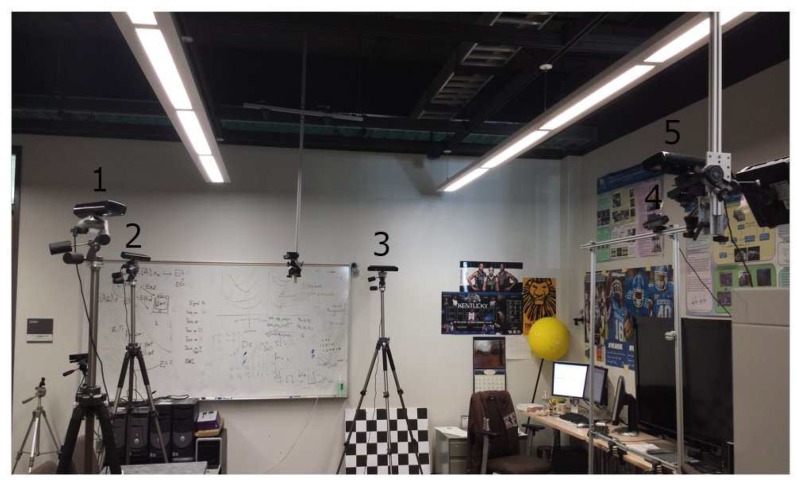
Overview of our camera network setup.

The setup of our camera network is shown in Figure 5. The camera network consists of 5 Kinect cameras sparsely placed in an indoor room of 45.36 m${}^{2}$. A client-server architecture is built for parallel computing and data collection from the cameras. Each camera is connected to a separate client computer, which is a Wintel machine with an Intel Core 2 Quad Q9650 processor, 8 GB of RAM, running Windows 7. The server is a Wintel machine with an Intel Core i7-5820k processor and GeForce GTX-1080 GPU, 32.0 GB of RAM, running Windows 10. The local network is a 100BASE-TX Ethernet. In practice, our proposed system does not require extra setup effort or additional equipment. We have tested a 5-camera network using Wi-Fi and commodity hardware in our laboratory, various classrooms and auditorium in our university. During the initial calibration stage, each client sends the identified sphere centers and timestamp information to the server. For online 3D rendering, each client sends aligned color and depth images with 640×480 resolution at 30 fps to the server. As such, static and dynamic objects are captured and reconstructed in real time. To ensure the server receives the accurate corresponding frames sent by each camera, we set up a local Network Time Protocol (NTP) server to synchronize all computers. The time server is equipped with a GPS board, which provides a precise PPS (Pulse Per Second) signal for time synchronization. After synchronizing with the local time server, the system time for capturing each frame among all computers is within a 4-ms offset.

The performance of the proposed system described in Section 3 is systematically measured. A yellow sphere with known radius $\bar{R}$ is used as the calibration object as described in Section 3.2. We have tested spheres of different radii ranging from 127 mm–203.2 mm. While there is no definitive mathematical relationship between the size of the ball and the algorithm, a larger ball is visible to more cameras and in general requires fewer video frames for calibration. As such, all the results in Section 3.2 are based on using the sphere with radius $\bar{R}=$ 203.2 mm. The three approaches of camera view transformation described in Section 3.3, including rigid transformation, polynomial regression and manifold alignment, were tested. For polynomial regression, we have tested the linear feature mapping feature and two variants of the quadratic feature mapping: a simplified version (14) without the cross terms and (14) itself. These three forms of regressions are denoted as Regression I with 12-DOF, Regression II with 18-DOF and Regression III with 30-DOF, respectively. Each of these methods is combined with the simultaneous optimization step as described in Section 3.4. To compare these methods with the state-of-the-art, we include the scheme by Herrera et al. [33] based on their publicly available software. Both quantitative and qualitative results of calibrations were measured as described below.

### 4.1. Quantitative Evaluation

For the initial calibration, about 1000 RGB-D images are captured for sphere detection by each camera. The whole calibration process is fully automatic in our constructed client-server architecture. The speed performance of each task during the calibration stage is shown in Table 1. The total execution time for our calibration is 47 s. The short execution time enables rapid reconfiguration of the camera network for any target applications. For real-time 3D capture after the calibration, the pairwise calibration step and simultaneous optimization are no longer needed. Instead, the color and depth data are locally compressed using motion-JPEG and transmitted to the server for rendering. The bandwidth requirement for 5 cameras is measured to be 157.4 Mbits per second on average, and the rendering speed of the point clouds from all cameras at the server is approximately 20 frames per second.

Next, we evaluate our sphere fitting algorithm. The goal of the sphere fitting algorithm is to estimate the location of the sphere center C(t) at frame *t* based on the observed 3D depth points $X_{d_{i}}\left(t\right)$ for $i=1,\ldots ,D_{p}\left(t\right)$ identified on the sphere surface. While it is difficult to establish the ground truth for the unobservable sphere centers, we know the ground truth radius of the sphere to be $\bar{R}=$ 203.2 mm. As such, we can calculate the average deviation from the ground truth radius of the radii estimated based on the identified sphere centers:

(20) $$\sigma =\sqrt{\frac{1}{\sum_{t=1}^{T}D_{p}\left(t\right)}\sum_{t=1}^{T}\sum_{i=1}^{D_{p}\left(t\right)}{\left(\bar{R}-∥X_{d_{i}}\left(t\right)-C\left(t\right)∥\right)}^{2}}$$

Three hundred sequential frames are tested to evaluate the sphere fitting accuracy. The estimate is unbiased with standard deviation σ equal to 6.42 mm or 3.15% of the ground truth radius.

**Figure 6:**
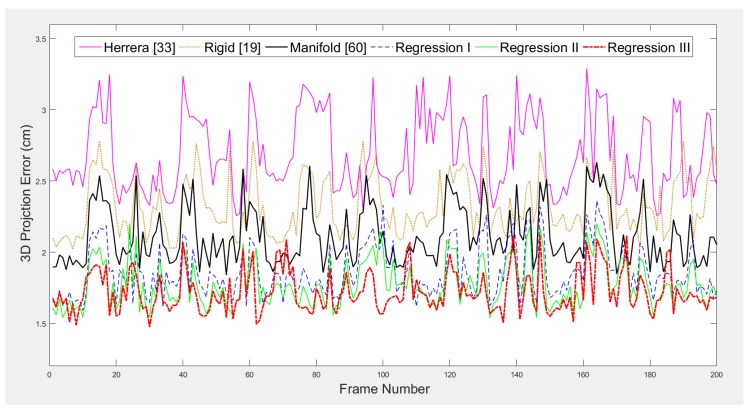
The 3D projection error of Camera 1 for each frame.

After the initial pairwise calibration, the acquired initial extrinsics are then optimized by our pseudo bundle adjustment algorithm. To test whether the estimated extrinsics can extrapolate unseen data, we apply them on a separate testing set of RGB-D images, which has the 200 detected sphere centers for each camera, to validate the correctness of the calibration. The back-projection error for each camera is compared across all the schemes. To calculate back-projection error, we use the pre-computed extrinsic matrices to project the 3D sphere center locations in local coordinates to the global frame, take the average of the projections from all the cameras, back-project it onto each local coordinate system and calculate the root mean square error (RMSE). To show that there is no bias in each method, we show the 3D projection error of Camera 1 for each frame in Figure 6. As shown in the figure, different curves representing different schemes seldom cross over each other. This shows that the relative performance among the five schemes stays constant regardless of the location of the sphere. As the plots for other cameras are similar, only the results for Camera 1 are shown in the figure. Table 2 shows the mean and standard deviation of the 3D projection errors of the entire trajectory for each camera-method combination. Table 3 shows the *p*-values when comparing Regression III with each the other methods, with the null hypothesis that the two methods produce similar results and the alternative hypothesis that Regression III produces better results. Based on the recommendation by Fisher [65], there is very strong evidence (p<0.001) among the majority of the cameras against the null hypothesis when comparing Regression III with Herrera [33], rigid [19] and manifold [60]. On the other hand, there is no evidence (p>0.1) against the null hypothesis when comparing Regression III with the other two regression techniques.

**Figure 7:**
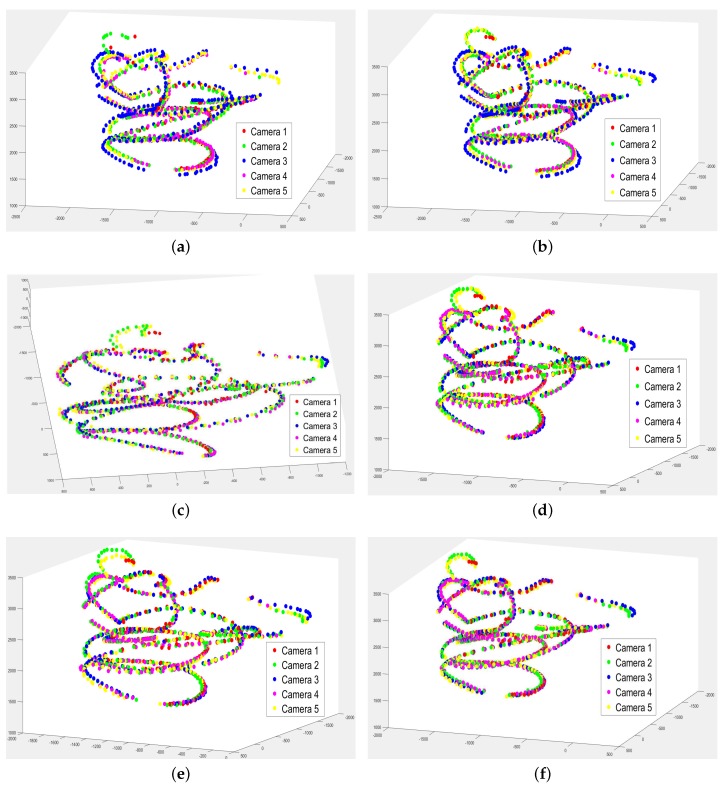
Sphere movement points alignment: (**a**) Herrera [33]; (**b**) rigid [19]; (**c**) manifold [60]; (**d**) Regression I; (**e**) Regression II; (**f**) Regression III.

For the visual alignments, the plots of the sphere movement points in the global frames are in Figure 7. One can see that there are significant misalignment error among trajectories from different cameras in Herrera’s scheme and the rigid scheme. The manifold scheme produces a skewed global frame, but appears to produce reasonable alignment. All regression schemes produce similar alignment results, with minor improvements as the degree of freedom increases. Table 4a–f shows the average differences in sphere center location between each camera pair with respect to Figure 7a–f. We should caution that while the testing data are different from the training data, they are all captured in the same center area where there is a significant overlap among the fields of view of different cameras. To extrapolate the coverage into areas with little overlap, we evaluate in the next section the visual quality of captured 3D environment including static background and moving foreground when the system performs real-time 3D rendering.

### 4.2. Qualitative Evaluation

**Figure 8:**
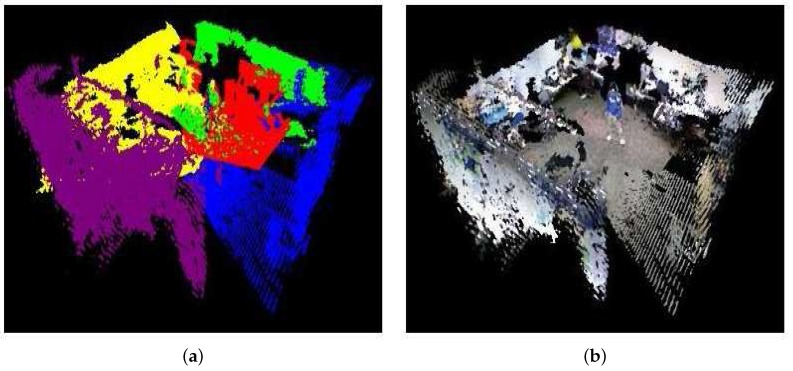
Real-time camera view merging in an indoor room using Regression I. The field of view for each camera is rendered in a different color in the left image.

**Figure 9:**
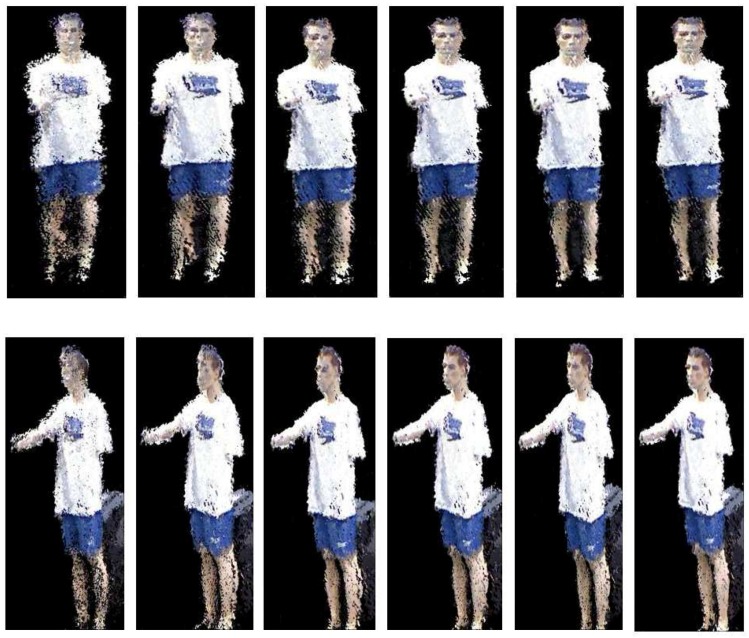

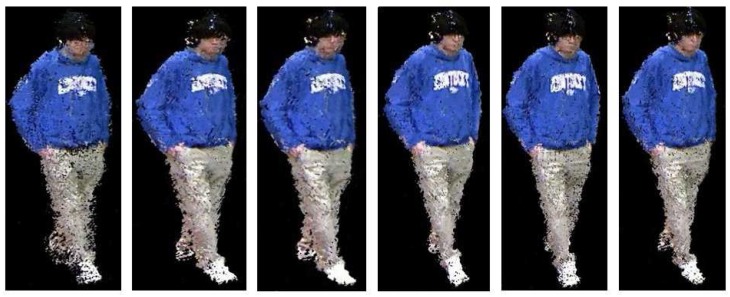
Comparison of 3D point cloud alignments zoom-in on the specific targets. From left to right column: (1) Herrera [33]; (2) rigid [19]; (3) manifold [60]; (4) Regression I; (5) Regression II; (6) Regression III.

**Figure 10:**
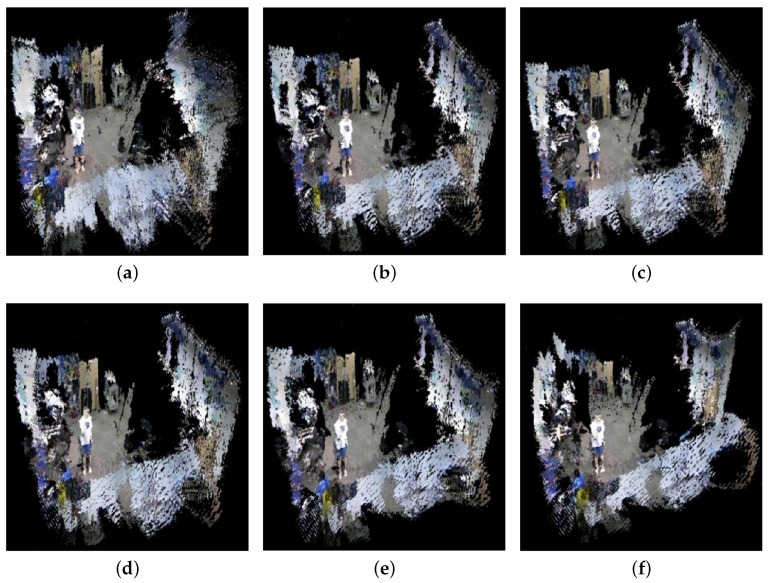
Comparison of 3D point cloud alignments on the indoor environment. From the top to the bottom (row-major order): (**a**) Herrera [33]; (**b**) rigid [19]; (**c**) manifold [60]; (**d**) Regression I; (**e**) Regression II; (**f**) Regression III.

In this section, we evaluate the results of our real-time 3D rendering in an indoor environment by the RGB-D camera network. Figure 8a,b shows the coverage area from each camera and the merged camera view, respectively. One can see that the center area has a higher density of point cloud than the surroundings. To compare alignment accuracy among different camera view transformations, we first consider the reconstruction of the foreground objects near the center of the captured area, which include a stationary mannequin and a walking person. Two randomly-selected virtual viewpoints of the mannequin and one viewpoint of the person are shown in Figure 9. Similar to the numerical results in Section 4.1, using polynomial regression produces better alignment on the model’s face and feet than the other methods. Next, we evaluate the reconstruction quality of the entire indoor scene for each view transformation method. In Figure 10a–f, Herrera’s scheme has a significant alignment problem when extending beyond the center area. The rigid and manifold schemes produce similar reconstruction results. All regression schemes have better alignments (less holes in the reconstructions), but Regression II and III start to introduce non-linear distortion in the background wall. The reason is the data from the training set are overfitted by higher degree parameters and cross terms, which can no longer preserve the geometry of the far-distance objects outside the calibration area. Overall, Regression I produce the best reconstruction results in our experiments with the minimal amount of misalignment and geometrical distortion.

## 5. Conclusions

In this paper, we have presented a fast and robust method for RGB-D camera network calibration using a spherical calibration object. The proposed 3D sphere center estimation scheme has been shown to produce a robust estimate of the sphere center trajectory. Compared with planar calibration objects, our solution is more reliable and requires less overlap in camera views. Using the observed sphere center trajectories at different camera views, we have tested four types of extrinsic camera calibrations including rigid transformation, manifold alignment, linear transformation and quadratic transformation, followed by a customized simultaneous refinement based on bundle adjustment. Our results have shown that linear transformation produced the best results by providing good alignment in the central view overlapping region and preserving geometric features in the peripheral area that has limited camera coverage. The proposed scheme has been implemented using a client-server architecture that enables rapid calibration and real-time 3D dynamic scene rendering. We are currently extending our scheme to cover an area as large as an entire building and to develop more data-efficient representations so as to scale the network to tens and hundreds of cameras. Furthermore, more investigations on mitigating distortion effects of metric structures of 3D space using polynomial approaches will be conducted in our future work. 

## Figures and Tables

**Table 1:** Execution performance on our RGB-D camera network calibration.

Task	Processing Time
Sphere center detection	44 ms (per frame)
Pairwise calibration	60 ms
Simultaneous optimization	2.2 s

**Table 2:** 3D projection errors on validation data.

Local Coordinate	Herrera [33]	Rigid [19]	Manifold [60]	Regression I	Regression II	Regression III
Camera 1	2.74 ± 0.23	2.40 ± 0.2	2.24 ± 0.22	1.98 ± 0.19	1.87 ± 0.17	1.80 ± 0.15
Camera 2	2.73 ± 0.22	2.36 ± 0.21	2.01 ± 0.23	2.01 ± 0.15	1.94 ± 0.16	1.88 ± 0.18
Camera 3	4.94 ± 0.54	4.56 ± 0.42	2.29 ± 0.22	2.12 ± 0.2	1.90 ± 0.16	1.85 ± 0.2
Camera 4	2.86 ± 0.22	2.29 ± 0.18	1.56 ± 0.12	1.44 ± 0.11	1.40 ± 0.1	1.49 ± 0.12
Camera 5	1.86 ± 0.17	2.33 ± 0.2	2.27 ± 0.17	2.05 ± 0.19	1.88 ± 0.17	1.84 ± 0.17
Average (cm)	3.03	2.79	2.07	1.92	1.80	1.77

**Table 3:** *p*-value for hypothesis testing.

Local Coordinate	R. III - Herrera [33]	R. III - Rigid [19]	R. III - Manifold [60]	R. III - R. I	R. III - R. II	R. II - R. I
Camera 1	0.0001	0.0001	0.0001	0.0905	0.9996	0.9999
Camera 2	0.0001	0.0001	0.9998	0.9952	0.9999	0.9999
Camera 3	0.0001	0.0001	0.0001	0.1302	0.9999	0.9999
Camera 4	0.0001	0.0001	0.0001	0.1517	0.9989	0.9999
Camera 5	0.0001	0.0001	0.0001	0.8743	0.9999	0.9999

**Table 4:** Average differences (cm) in sphere location between each camera pair over all frames.

(a) Herrera [33]							(b) Rigid [19]					
**Camera**	***C*****_1_**	***C*****_2_**	***C*****_3_**	***C*****_4_**	***C*****_5_**		**Camera**	***C*****_1_**	***C*****_2_**	***C*****_3_**	***C*****_4_**	***C*****_5_**
*C*_1_	∖	2.98	5.39	3.08	3.01		*C*_1_	∖	2.36	5.02	2.57	3.03
*C*_2_	2.98	∖	5.69	2.85	2.77		*C*_2_	2.36	∖	5.57	2.32	2.95
*C*_3_	5.39	5.69	∖	6.05	4.73		*C*_3_	5.02	5.57	∖	5.89	4.52
*C*_4_	3.08	2.85	6.05	∖	2.46		*C*_4_	2.57	2.32	5.89	∖	2.99
*C*_5_	3.01	2.77	4.73	2.46	∖		*C*_5_	3.03	2.95	4.52	2.99	∖
(c) Manifold [60]							(d) Regression I					
**Camera**	***C*****_1_**	***C*****_2_**	***C*****_3_**	***C*****_4_**	***C*****_5_**		**Camera**	***C*****_1_**	***C*****_2_**	***C*****_3_**	***C*****_4_**	***C*****_5_**
*C*_1_	∖	2.42	2.67	2.33	2.75		*C*_1_	∖	2.23	2.64	2.0	2.46
*C*_2_	2.42	∖	3.02	2.48	2.39		*C*_2_	2.23	∖	2.37	2.07	2.25
*C*_3_	2.67	3.02	∖	2.33	2.95		*C*_3_	2.64	2.37	∖	2.07	2.34
*C*_4_	2.33	2.48	2.33	∖	2.0		*C*_4_	2.0	2.07	2.07	∖	1.79
*C*_5_	2.75	2.39	2.95	2.0	∖		*C*_5_	2.46	2.25	2.34	1.79	∖
(e) Regression II							(f) Regression III					
**Camera**	***C*****_1_**	***C*****_2_**	***C*****_3_**	***C*****_4_**	***C*****_5_**		**Camera**	***C*****_1_**	***C*****_2_**	***C*****_3_**	***C*****_4_**	***C*****_5_**
*C*_1_	∖	2.27	2.6	1.95	2.26		*C*_1_	∖	2.19	2.43	2.05	2.24
*C*_2_	2.27	∖	2.43	2.17	2.24		*C*_2_	2.19	∖	2.38	2.11	2.13
*C*_3_	2.6	2.43	∖	2.07	2.18		*C*_3_	2.43	2.38	∖	2.27	2.17
*C*_4_	1.95	2.17	2.07	∖	1.7		*C*_4_	2.05	2.11	2.27	∖	1.75
*C*_5_	2.26	2.24	2.18	1.7	∖		*C*_5_	2.24	2.13	2.17	1.75	∖

## Supplementary Materials

The source codes for our RGB-D camera network calibration are available online at https://github.com/PoChang007/RGBD_CameraNetwork_Calibration.
